# Exploring the effects of adolescent social isolation stress on the serotonin system and ethanol-motivated behaviors

**DOI:** 10.1007/s00213-025-06749-3

**Published:** 2025-02-04

**Authors:** Bryan D. McElroy, Chen Li, Nicholas S. McCloskey, Amber R. Alberici, Lynn G. Kirby

**Affiliations:** Center for Substance Abuse Research, Lewis Katz School of Medicine at Temple University, 3500 N. Broad St, MERB Room 857, Philadelphia, PA 19140 USA

**Keywords:** Sex differences, Punishment-resistant drinking, Early life stress, Ethanol operant conditioning, Electrophysiology, DREADDs, Chemogenetics, Tph2-iCre, Serotonin, Rats

## Abstract

**Rationale:**

Alcohol is one of the most frequently used drugs of abuse and has a major impact on human health worldwide. People assigned female at birth and those with adverse childhood experiences are stress-vulnerable and more likely to report drinking as a means of “self-medication.” Prior studies in our laboratory showed that adolescent social isolation stress (SIS) increases vulnerability to ethanol (EtOH) intake and consumption despite negative consequences in female rats.

**Objectives:**

Here, we explored modulation of the dorsal raphe nucleus (DRN)-serotonin (5-HT) system, a sexually dimorphic neurotransmitter system involved in stress-reward interactions, to determine its contribution to EtOH-motivated behaviors in rats that have undergone SIS.

**Results:**

We employed electrophysiological and functional neuroanatomy strategies to show that both SIS and EtOH exposure induce persistent hypofunction of the DRN 5-HT system, particularly in females. Chemogenetic activation of DRN 5-HT neurons attenuated reward value for both EtOH and sucrose and elevated punished responding for EtOH in a stress-dependent manner.

**Conclusions:**

Our results highlight an inverse relationship between EtOH consumption and the 5-HT system, the sex- and stress-dependent nature of this relationship, and a connection between DRN 5-HT signaling and acute responding to rewards and punishment. These data support the DRN 5-HT system as a potential target to treat aberrant alcohol consumption and drinking despite negative consequences in stress-vulnerable populations.

**Supplementary Information:**

The online version contains supplementary material available at 10.1007/s00213-025-06749-3.

## Introduction

Excess or frequent alcohol use is the seventh leading risk factor for premature death and disability around the world (National Institute on Alcohol Abuse and Alcoholism 2023b). Recently, a goal of the World Health Organization has been to identify a potential “safe” level of alcohol consumption without risk of illness or injury (World Health Organization 2023). The National Institute on Alcohol Abuse and Alcoholism defines low-risk, moderate drinking as no more than 1 or 2 standard drinks a day for females and males, respectively (National Institute on Alcohol Abuse and Alcoholism 2023a). However, about 2 in 100 people who drink within these limits meet the criteria for alcohol use disorder (AUD) and may experience adverse effects if they drink too quickly or have other health-related issues and vulnerabilities (National Institute on Alcohol Abuse and Alcoholism 2018).

One such vulnerability is early life stress (ELS). Clinical and preclinical studies show that ELS, such as abuse, neglect, and trauma, can profoundly affect mood and behavior in adulthood, resulting in a strong correlation between adverse childhood experiences, compulsive drug use, and psychological distress (Fenton et al. 2013; Gershon et al. 2013; Kirsch et al. 2020; Manyema et al. 2018). Although much remains to be understood about the possible pathophysiology of ELS-induced alcohol consumption, several candidate systems and processes have been identified, such as dysfunction in stress and reward brain regions, including their regulation by the serotonin system (Enoch 2011; Kirby et al. 2011; Koob and Volkow 2016; Underwood et al. 2018).

The dorsal raphe nucleus (DRN), the largest of the serotonergic nuclei, projects to both cortical and subcortical brain regions to regulate a diversity of functions, including mood, stress, and reward (Commons 2020; Okaty et al. 2019). The 5-HT system is extremely complex and diverse, showing sexual dimorphism as well as physiological and molecular heterogeneity (Calizo et al. 2011; Commons 2020; Okaty et al. 2019; Sharp and Barnes 2020). There is a large body of evidence that consistently shows that both chronic stress and excessive alcohol consumption have an inverse relationship with 5-HT (LeMarquand et al. 1994a, b; Marcinkiewcz 2015; Marcinkiewcz et al. 2016). Clinically, subjects with AUD have shown low levels of the 5-HT metabolite 5-HIAA in their cerebrospinal fluid (Borg et al. 1985; Fils-Aime et al. 1996). This is echoed in the preclinical literature in which alcohol-preferring rats have deficits in basal 5-HT levels (Chastain 2006), and 5-HT-deficient transgenic mice show elevated ethanol (EtOH) consumption compared to wild-type controls (Sachs et al. 2014). However, acute EtOH consumption transiently elevates extracellular levels of 5-HT in multiple brain regions (see review (Kirby et al. 2011), an effect exacerbated in 5-HT deficient, alcohol-preferring rats. This suggests that alcohol consumption may be a means of “self-medication” to normalize hypofunction of the 5-HT system in at-risk populations. This idea is also consistent with the “opponent process” theory in which drug taking can be triggered by “drug opposite” states, including hypofunctioning reward systems and negative mood states (e.g., dysphoria, anhedonia, anxiety) (Koob and Moal 1997; Solomon and Corbit 1974). In this model, drug taking is an attempt to alleviate this discomfort and thus is a form of “self-medication” (Markou et al. 1998).

A recent study in our laboratory investigated the effects of sex and ELS on EtOH-motivated behaviors in adulthood utilizing the ethologically relevant adolescent social isolation stress (SIS) paradigm (McElroy et al. 2023). The existing literature examining SIS effects on EtOH consumption in rats has shown mixed effects that depend on the strain and sex as well as the SIS regimen and the drinking model employed (Chappell et al. 2013; Butler et al. 2014; Skelly et al. 2015; Ortelli et al. 2023; Deal et al. 2021; Pitcairn et al. 2024). Our results using a model of moderate drinking in Sprague-Dawley rats showed SIS-induced elevations in EtOH preference, intake, and consumption despite negative consequences, particularly in females (McElroy et al. 2023). These sex- and stress-dependent effects are particularly salient as they were observed in a moderate or “social drinking” model (McElroy et al. 2023). The goal of the current study is to expand upon our previous work to investigate the role of the DRN 5-HT system in these sex- and stress-dependent effects on EtOH-motivated behaviors in adulthood. After establishing baseline electrophysiology profiles in males and females, this study focused primarily on the stress-vulnerable female group.

### Disclaimer

All cited clinical references were conducted in cisgender women and men unless otherwise stated and may not necessarily translate to intersex, nonbinary, transgender, and gender-nonconforming individuals.

## Materials and methods

### Subjects

**Fig. 1:**
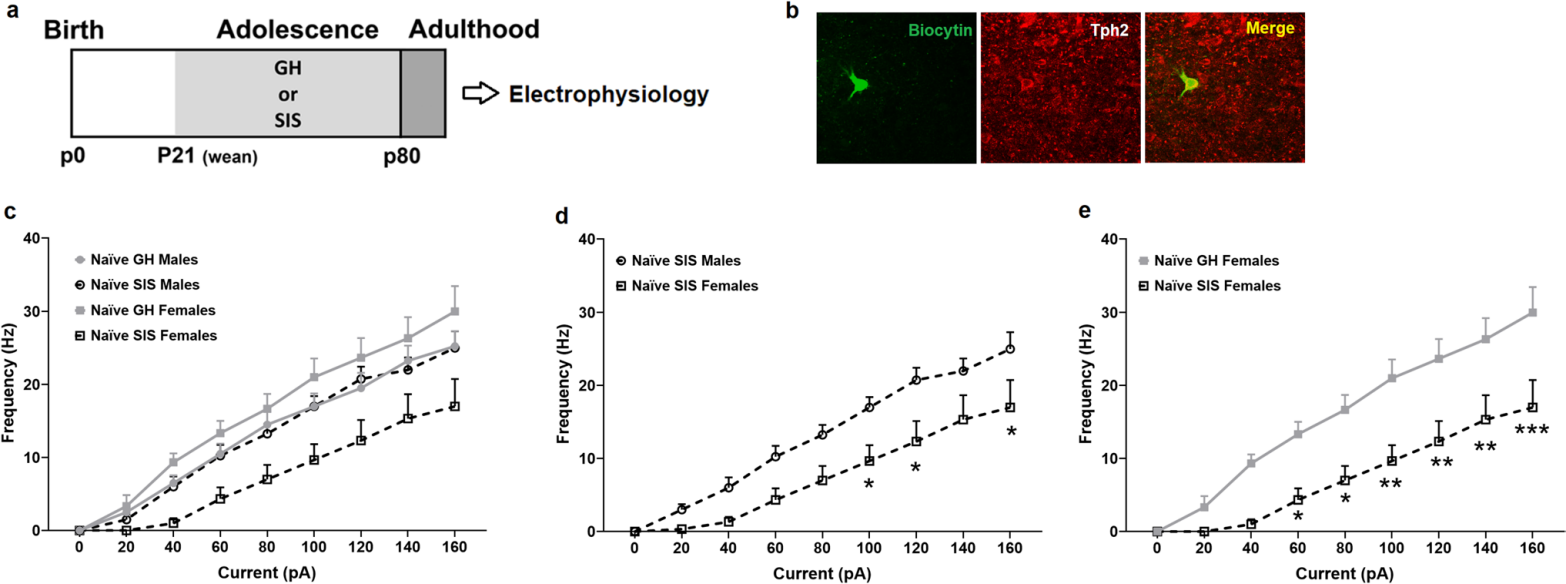
Excitability of DRN 5-HT neurons in male and female EtOH-naïve rats. **a** Experimental timeline. **b** Post hoc immunohistochemical identification of Tph2 recorded neurons, biocytin-filled recorded neuron (green) and Tph2 + neurons (red) is shown. SIS females showed hypoexcitability of DRN 5-HT neurons in adulthood relative to other treatment groups (**c**), vs. male SIS at currents of 100, 120 and 160pA (**d**) and vs. female GH controls at currents of 60-160pA (**e**). Asterisks indicate significant differences by post hoc Sidak tests (*p < 0.05, **p < 0.01, ***p < 0.001). Total *n* = 28 cells. GH males *n* = 8 (solid gray circles), SIS males *n* = 8 (open black circles), GH females *n* = 6 (solid gray squares), SIS females *n* = 6 (open black squares)

**Fig. 2:**
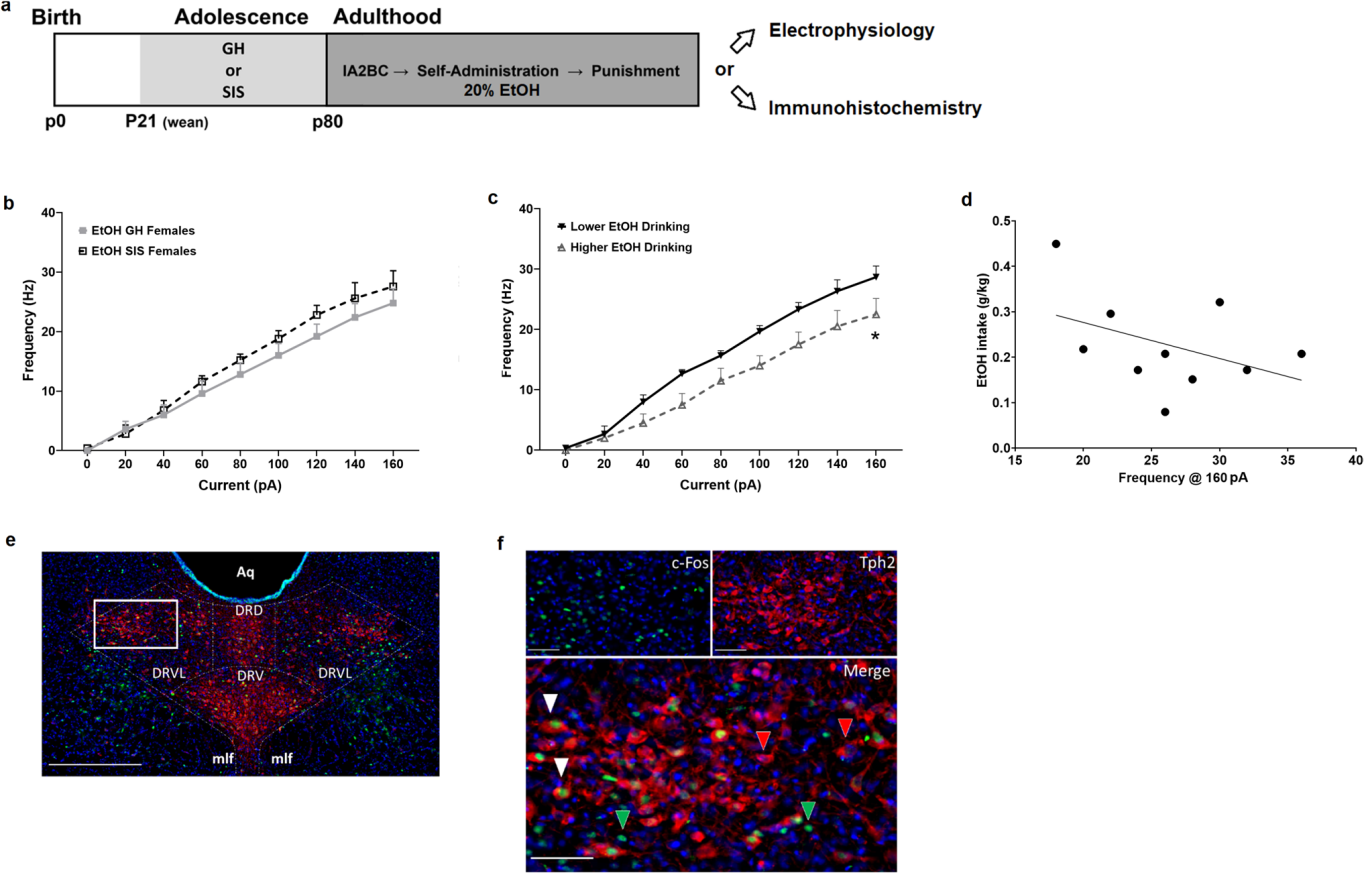
Excitability and c-Fos expression of DRN 5-HT neurons in female EtOH-exposed rats. **a** Experimental timeline. **b** When split by stress, no significant differences were observed between treatment groups. **c** When median-split by EtOH intake over the last three self-administration sessions, higher drinking rats showed significant hypoexcitability of DRN 5-HT neurons compared to lower drinking rats. **d** However, the correlation between individual EtOH intake and excitability of DRN 5-HT neurons did not reach statistical significance. Asterisks indicate significant differences by post hoc Sidak test (*p < 0.05). Total *n* = 10 cells. a; GH females *n* = 5 (solid gray squares), SIS females *n* = 5 (open black squares). b; lower drinking *n* = 6 (solid black upside-down triangles), higher drinking *n* = 4 (open gray triangles). **e** and **f** Representative photomicrographs of immunofluorescence staining in the DRN following punished SA. Images show staining of Tph2 (red), c-Fos (green), and DAPI (blue). **e** Merged image at 20x magnification outlining the locations of the different DRN subdivisions. **f** Higher magnification images of the framed area in panel **e**. The images show (from top left to bottom right) staining of c-Fos, Tph2, and a merged image, respectively. Arrowheads in the merged image indicate cells that are single-labeled for c-Fos (green) or Tph2 (red), as well as cells with dual expression (white). Scale bars indicate 500 μm and 75 μm for images in panels **e** and **f**, respectively. Aq = cerebral aqueduct, DRN = dorsal raphe nucleus, DRD = dorsal DRN, DRV = ventral DRN, DRVL = ventrolateral DRN and mlf = medial longitudinal fasciculus

**Fig. 3:**
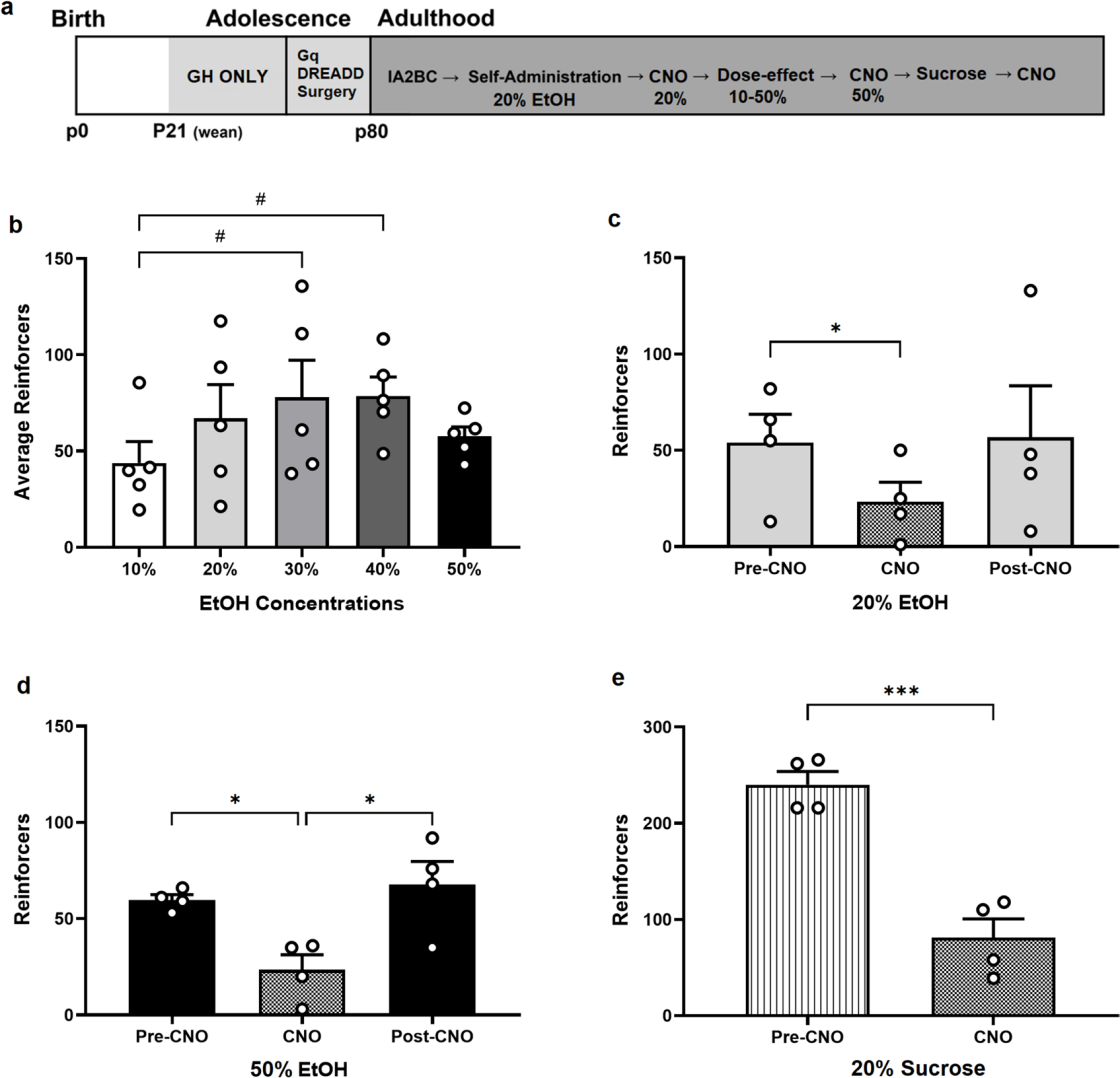
The effect of Gq-DREADD activation of DRN 5-HT neurons on reinforcers earned during EtOH and sucrose self-administration in GH female rats. **a** Experimental timeline. **b** EtOH dose-effect curve showing the average reinforcers earned at escalating concentrations of EtOH, with peak responding at 30–40%. CNO activation of Gq DREADDs significantly reduced reinforcers earned for 20% EtOH (**c**), 50% EtOH (**d**) and 20% sucrose (**e**). Pound signs indicate significant differences by post hoc Tukey tests (#*p* < 0.05). Asterisks indicate significant differences by two-tailed paired t-tests (*p < 0.05, ***p < 0.001). Total GH females *n* = 5 (**b**) and *n* = 4 (**c**-**e**)

**Fig. 4:**
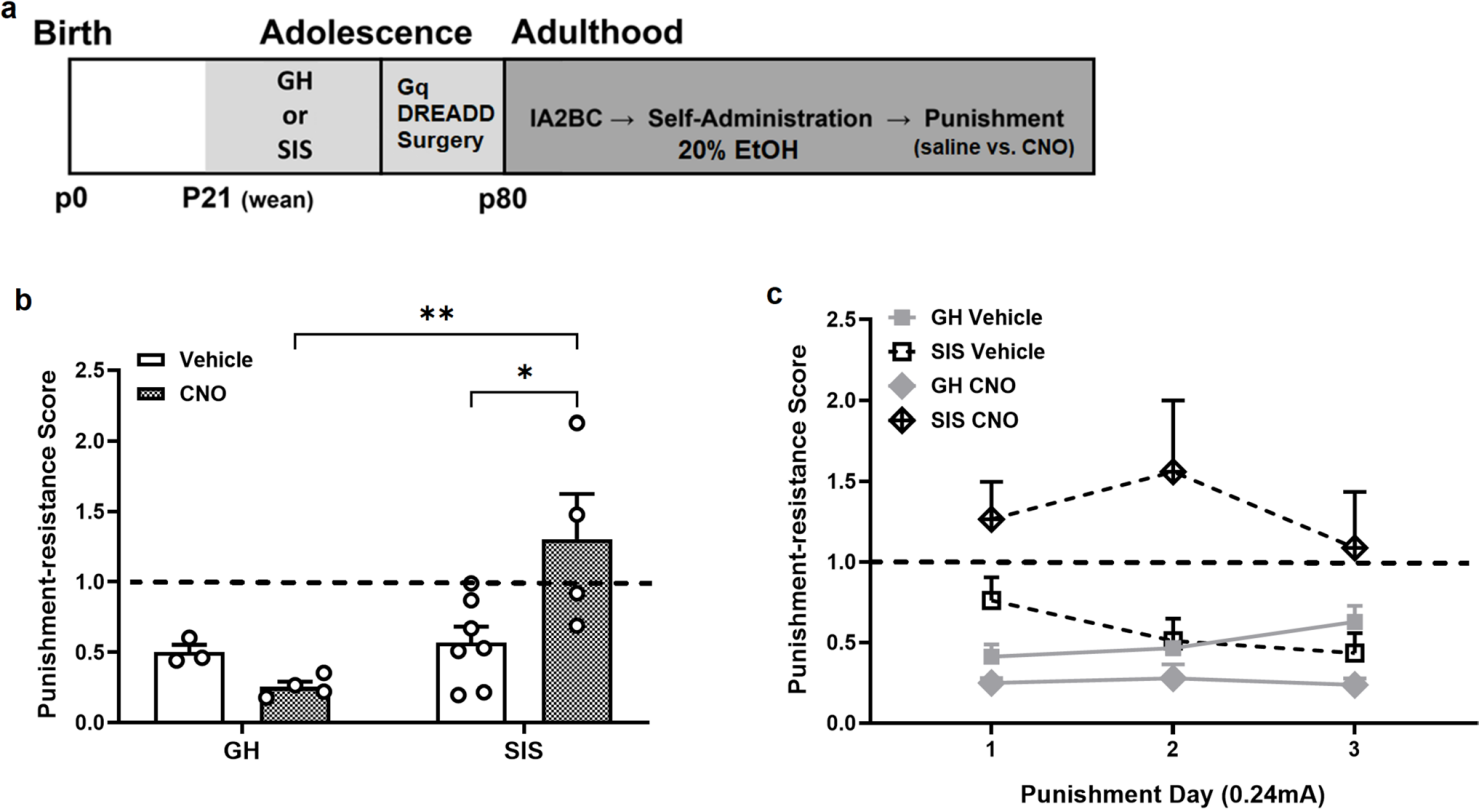
The effect of Gq-DREADD activation of DRN 5-HT neurons on footshock-punished EtOH SA in female rats. CNO activation of Gq DREADDs produced stress-specific effects on punished EtOH SA. **a** Experimental timeline. **b** CNO elevated average punishment resistance scores in SIS but not GH rats. **c** Punishment resistance scores over three days of punished SA revealed that CNO elevated punishment resistance in SIS rats and reduced punishment resistance in GH rats compared to their respective vehicle controls. Asterisks indicate significant differences by post hoc Sidak tests (*p < 0.05, **p < 0.01). Total rats *n* = 18, GH vehicle *n* = 3, GH CNO *n* = 4, SIS vehicle *n* = 7, SIS CNO *n* = 4

Male and female transgenic Tph2-iCre rats (hemizygous and wild-type littermates on a Sprague-Dawley background) were bred in-house at Temple University, derived from breeders generously provided by Drs. Katinka Stecina and Larry Jordan at the University of Manitoba, Canada, originally developed by Drs. Dusan Bartsch and Kai Schoenig (Weber et al. 2011). This transgenic strain was used to investigate the role of the serotonin system in the sex- and stress-dependent nature of EtOH consumption. Hemizygous Tph2-iCre rats were validated by immunohistochemical analysis, demonstrating nearly 100% colocalization of Tph2-immunoreactivity with Cre recombinase-immunoreactivity (Li et al. 2025). Pilot studies in our laboratory have indicated similar drug self-administration behavior in this transgenic strain when compared to commercially obtained Sprague-Dawley rats (Taconic Biosciences, Germantown, NY), indicating that the behavioral phenotype of this rat strain reflects its genetic background. For electrophysiological studies conducted in EtOH-naïve rats, both male and female rats were used; all other experiments were conducted solely in female rats. All rats were housed on a reverse 12-hour light/dark cycle (lights on at 21:00, lights off at 09:00) and had *ad libitum* access to standard lab chow and water throughout all studies. A total of 71 rats from 18 litters were used for the study, and pups from each litter were randomly assigned to different experimental groups in order to control for litter effects. Behavioral testing was performed during the rat dark cycle. Experimental timelines are shown in Figs. 1, 2, 3 and 4. All procedures were conducted in accordance with the policies set forth by Temple University Institutional Animal Care and Use Committee and the National Research Council’s *Guide for the Care and Use of Laboratory Animals.*

### Adolescent social isolation stress (SIS)

As described previously (McElroy et al. 2023), rats were weaned on postnatal day (p) 21 and randomly assigned to either same-sex pair housing (group housed, GH), or single housing (SIS) conditions (Haj-Mirzaian et al. 2019; Mumtaz et al. 2018; Song et al. 2021). Rats remained untouched during adolescence (p21-p60) with the exception of biweekly cage changes by Temple University husbandry staff. All rats were then single-housed on p60 and remained so for the rest of the study.

### EtOH- and sucrose-exposure paradigms

Most drugs of abuse including µ-opioid agonists and psychostimulants are readily self-administered in rodents (Green and Grahame 2008). However, rodents can initially find EtOH aversive. Therefore, at p80, we introduced rats to 20% EtOH *(v/v)* in their homecage in an intermittent access two-bottle choice (IA2BC) model as described previously (Carnicella et al. 2014; McElroy et al. 2023; Pirino et al. 2022; Simms et al. 2008). Briefly, at 09:00 in the beginning of the dark cycle, rats were given 20% EtOH (v/v) and one bottle of water for 24 h every other day, 3 days a week (Monday, Wednesday, Friday) for 4 weeks. This entices subsequent EtOH SA (Carnicella et al. 2011) and avoids having to use a sucrose-fade model of EtOH SA, which may have confounds resulting from the natural rewarding effects of sucrose (Jeanblanc et al. 2019). Following homecage drinking (4 weeks), EtOH-exposed rats were transferred to oral operant EtOH SA protocol as described previously (McElroy et al. 2023) using rat operant SA chambers in sound-attenuating cubicles (Med Associates, St Albans, VT). This EtOH SA protocol employs a small 0.01 mL dipper and is intended to model moderate rather than excessive drinking, eliciting blood ethanol concentrations less than 80 mg/dl with relevance to “social drinking” (1–3 drinks in humans) (McElroy et al. 2023). A separate cohort of age-matched rats were exposed to sucrose SA. Rats underwent 2 days of 2-hour magazine training sessions during which 20% EtOH *(v/v)* or 10% sucrose *(w/v)* was presented every 6 min. Following magazine training, rats were shifted to 2-hour oral EtOH or sucrose SA on a fixed ratio 1 schedule of reinforcement, 5 days a week until they met acquisition criteria (average reinforcers during last 3 days of SA ≥ 15% (Bertholomey et al. 2016; Domi et al. 2021); approximately 15 days). During SA, rats were presented with 2 levers: one active lever and the other inactive (responding yielded no consequences). Active lever responding produced a 2-second light-tone (75 dB) cue, simultaneous with the raising of a 0.01 ml dipper of EtOH or sucrose in the magazine (reinforcer) for 22 s. Following the presentation of the reinforcer, there was a 20-second timeout period in which active lever responding did not result in an EtOH or sucrose delivery. To increase the accuracy of intake during SA, we counted only reinforcers with confirmed magazine entries within the 22-second dipper availability period (Stafford et al. 2015). Following EtOH or sucrose SA, rats underwent 3 days of footshock-punished SA. Punishment sessions were identical to SA, except 50% of reinforcers were paired with a mild 0.24 mA footshock (Marchant et al. 2013). The footshock was delivered for 0.5 s concurrent with the 2-second light-tone cue, which was only activated upon earning a reinforcer. Cues were absent during the remainder of the 22-second dipper presentation.

### Electrophysiology

Electrophysiology recordings (Beck et al. 2004; Kirby et al. 2008) in EtOH-naïve GH and SIS male and female rats occurred at 15 weeks old, in line with the age of rats at the end of behavioral studies (McElroy et al. 2023). Recordings in EtOH-exposed GH and SIS female rats were done 90 min following their last punished SA session. Subjects were euthanized by rapid decapitation, and brains were placed into ice-cold artificial cerebrospinal fluid (ACSF), in which sucrose (248 mM) was substituted for NaCl. Slices (250 µm thick) were cut through the DRN using a Vibratome 3000 Plus (Vibratome, Bannockburn, IL) and placed in ACSF ((in mM): 124 NaCl, 3.0 KCl, 1.25 NaH_2_PO_4_, 2.5 CaCl_2_, 2 MgSO_4_, 10 dextrose, and 26 NaHCO_3_) with _L_-tryptophan (50 mM) at 35 °C bubbled with 95% O_2_/5% CO_2_ for 1-hour. Slices were then maintained at room temperature in ACSF bubbled with 95% O_2_/5% CO_2_.

Slices were transferred to a recording chamber (Warner Instruments, Hamden, CT) and continuously perfused with ACSF at 1.5–2.0 ml/min. Only one cell was recorded per brain slice. DRN neurons were visualized using a Nikon E600 upright microscope (Optical Apparatus, Ardmore, PA). Slices were then maintained at room temperature ACSF bubbled with 95% O_2_/5% CO_2_. The resistance of the electrode was 4–6 MΩ when filled with an intracellular solution ((in mM):120 K gluconate, 10 KCl, 2 MgCl_2_, 10 EGTA, 10 HEPES, 2 MgATP, 10 NaPhosphocreatine, 0.5 Na_2_GTP, 0.1% biocytin, pH 7.3).

Neuronal excitability was assessed by whole-cell patch-clamp recordings under current-clamp conditions with a HEKA patch-clamp EPC-10 amplifier (HEKA Elecktronik Lambrecht/Pfalz, Germany). Excitability was assessed by voltage responses to a series of current pulses (−100 to 160pA, 500ms duration, step + 20pA). The neurochemical content of recorded cells was determined by post hoc dual fluorescence immunohistochemistry (IHC) with labeling of cells that are biocytin-filled [streptavidin Alexa 488, cat: S11223, donkey anti-rabbit, 1:1000] (Life Technologies, Carlsbad, CA) and Tph2-immunoreactive (primary: [cat: ABN60, rabbit anti-Tph2, 1:500] (MilliporeSigma, Burlington, MA) and secondary: [Alexa 568, cat: A10042, donkey anti-rabbit, 1:1000] (Life Technologies, Carlsbad, CA) (Kirby et al. 2008).

Additional membrane characteristics measured in recorded DRN 5-HT neurons (Table 1) include resting membrane potential (RMP), input resistance (measured from the voltage-current plot of each cell), action potential (AP) threshold, the change in voltage from RMP to AP threshold, the latency to AP initiation following DRN 5-HT neuron stimulation, AP amplitude (voltage measured from threshold to peak), AP duration (measured at threshold), AP rise time (time from threshold to peak) and AP decay time (time to return from peak to threshold).

Electrophysiological data were excluded from the study if recorded cells could not be conclusively identified as Tph2-positive by IHC. Cells from EtOH-exposed rats were excluded if rats did not meet SA acquisition criteria. To determine statistical outliers for electrophysiological studies, cells exceeding 2 standard deviations above or below the mean of their stress treatment groups for any of the cell characteristic endpoints were excluded from their respective data analyses. Cell outlier exclusion in EtOH-naïve rats was 2/10 GH males, 1/9 SIS males, 2/8 GH females, 2/8 SIS females. For EtOH-exposed rats, in addition to cell characteristics, cells from rats exceeding 2 standard deviations above or below mean EtOH intake (g/kg body weight) were excluded from data analyses. Cell outlier exclusion in EtOH-exposed rats was 4/14.

### Immunohistochemistry (IHC)

Ninety minutes following 20% EtOH- or 10% sucrose-punishment testing, female rats not used for electrophysiology studies were transcardially perfused, and brains were collected for IHC. Transcardial perfusions were performed using chilled phosphate-buffered saline (PBS) solution followed by 4% paraformaldehyde. Brains were then extracted and stored at 4 °C in 4% paraformaldehyde for 24 h before being transferred to a 30% sucrose solution containing 0.1% sodium azide (NaN_3_). Brains were flash frozen, and coronal sections (40 μm) containing the DRN were collected using a cryostat (Thermo Fisher Scientific Cryostar NX50, Waltham, MA) at −20 °C.

IHC for c-Fos was performed on free-floating brain tissue in 6 well plates stored in PBST (0.1% triton-x, 0.02% NaN_3_ in PBS) at 4 °C. All steps were performed at room temperature (20 °C), washes were done on a shaker for 10 min. Tissue was first washed (3x) before slices were transferred to primary antibody for c-Fos [cat: ABE457, rabbit anti-c-Fos, 1:2000] (MilliporeSigma, Burlington, MA) and Tph2 [cat: ab121013, goat anti-Tph2, 1:500] (Abcam, Cambridge, UK) in PBST and incubated on a shaker for 48 h at 4 °C. Sections were then washed (3x) in PBST before being transferred to c-Fos secondary antibody [Alexa 488, cat: A21206, donkey anti-rabbit, 1:1000] (Invitrogen, Carlsbad, CA) and Tph2 secondary antibody [Alexa 647, cat: A21447, donkey anti-goat, 1:1000] (Invitrogen, Carlsbad, CA) in donkey blocking solution (5% donkey serum stock (Sigma-Aldrich, St. Louis, MO), 0.3% triton-x, 0.1 M glycine, 0.02% NaN_3_ in PBS) and incubated on a shaker for 24 h at 4 °C. Following another wash (3x), tissue was wet-mounted to microscope slides and secured with coverslips using Fluoro-Gel II with Dapi mounting medium (Electron Microscopy Sciences, Hatfield, PA).

### Imaging and cell quantification

For all imaging and counting procedures, experimenters were blind to treatment groups. Tissue sections were imaged using a Keyence BZ-X800 (Keyence, Osaka, Japan) microscope with 20x magnification. Whole-image stitching was taken across the tissue section and used to determine colocalization of Tph2 and c-Fos. DRN sections used for IHC analyses were obtained from slices approximately − 7.6 to −8.00 mm posterior to bregma of the skull, where the DRN is the largest and includes the ventrolateral subdivision (DRVL) (Paxinos and Watson 2005). This aligns with other laboratories quantifying DRN c-Fos expression (Gardner et al. 2005; Greenwood et al. 2003; Hale et al. 2008). Cells were quantified in the following DRN subregions: dorsal subdivision (DRD), ventral subdivision (DRV), and the ventrolateral subdivision (DRVL) (Commons 2020; Lowry et al. 2008; Okaty et al. 2019). Quantification for the DRVL was determined as the sum of both the left and right sides of the subnucleus (Hale et al. 2008; Yamashita et al. 2017). For each subregion, 1–4 images were quantified for each animal. Identification and quantification of positive immunofluorescent staining was determined using BZ-X800 Analyzer, Hybrid Cell Count software (Keyence, Osaka, Japan). Quantifications were reported in both cell counts as well as percentages of the total number of cells to account for natural variations in DRN subdivision sizes in each slice. Endpoints included the total number of c-Fos-positive cells, the total number of Tph2-positive cells, the number of c-Fos/Tph2 colocalized neurons, the percentage of Tph2-positive neurons out of all c-Fos-positive neurons, and the percentage of c-Fos-positive neurons out of all Tph2-positive neurons. Tissue from rats that did not meet acquisition criteria for either sucrose or EtOH SA were excluded from IHC analyses (sucrose 1/11).

### Chemogenetic procedures

Prior to behavioral testing in adulthood, GH and SIS female rats hemizygous for Tph2-iCre received surgeries for intra-DRN viral delivery of Cre-dependent excitatory designer receptors exclusively activated by designer drugs (DREADD)s (AAV8-hSyn-DIO-hM3D(Gq)-mCherry; 3 × 10^12^ vg/ml; Addgene, Watertown, MA) as previously described (Roth 2016). Using standard aseptic technique, adult rats were anesthetized (ketamine (40 mg/kg)/xylazine (8 mg/kg) mixture, i.m.) and given the analgesic meloxicam (2 mg/kg, s.c.) before being placed into the stereotaxic apparatus. Rats received an infusion of the viral vector (2 µl volume at a rate of 0.2 µl/min (adapted from (Urban et al. 2016)) at the following coordinates: −7.8 mm caudal to bregma, − 2.2 mm from the midline and − 4.6 mm ventral from the brain surface at a 26° angle to bypass the sagittal sinus (Paxinos and Watson 2005). Rats were given up to 3 weeks of recovery to allow for viral transduction before being administered tamoxifen (40 mg/kg, i.p.) (Sigma-Aldrich, St. Louis, MO) once daily for 5 days to induce Cre-recombination (adapted from (Weber et al. 2011)). Following behavioral experiments, DRN sections were imaged by Leica Sp5 Confocal Microscope (Leica Microsystems, Exton, PA) for histological verification of mCherry fluorescence in the target area. Rats without mCherry expression, inflammation, or off-target expression were excluded from behavioral analysis or transferred to control groups (*n* = 3/18). Ex vivo slice electrophysiology was used to functionally validate excitatory Cre-dependent DREADDs in Tph2-iCre rats. After tamoxifen induction and preparation of brain slices containing the DRN, bath application of the DREADD ligand, clozapine-N-oxide (CNO) (10 µM) depolarized and induced firing in mCherry-expressing DRN 5-HT neurons (Li et al. 2025).

### Chemogenetic DRN 5-HT activation during EtOH & sucrose SA

Chemogenetic SA procedures were conducted within-subjects solely in GH female rats to evaluate DRN 5-HT neuron stimulation on voluntary reward behavior without the added variable of early life stress. Once EtOH responding was stabilized (3 consecutive days without a 15% change in reinforcers, adapted from (Bertholomey et al. 2016; Domi et al. 2021), rats were pretreated with CNO (2 mg/kg, i.p.) 30 min prior to 20% EtOH SA in order to activate Gq excitatory DREADDs in DRN 5-HT neurons. Following the DREADD activation experiment, rats returned to 20% EtOH SA until baseline responding (average reinforcers over last 3 days of SA before CNO treatment) was reestablished. Next, rats were introduced to escalating concentrations of EtOH from 10 to 50% for 2–3 days each. Responding at each EtOH concentration for the dose-effect curve (DEC) was taken from the average of 2–3 days of SA for each concentration. To test the effect of Gq DREADDs on the descending limb of our EtOH DEC, rats were treated with CNO, as previously described, prior to 50% EtOH SA. Following 50% EtOH SA, rats returned to baseline responding for 20% EtOH. Rats were left in the housing room for 2 weeks before being introduced to 20% *(w/v)* sucrose SA to evaluate natural reward. A previous study showed that 20% sucrose *(w/v)* is on the descending limb of the sucrose dose-effect curve in rats (Lê et al. 2006). The sucrose SA procedure was identical to EtOH SA, resulting in rapid acquisition and responding. The effect of chemogenetic activation of DRN 5-HT neurons on sucrose SA was evaluated as described above for EtOH. A total of 5 GH female rats were included in the EtOH DEC; however, only 4 rats were included in the chemogenetic SA endpoints. This rat was excluded from chemogenetic studies due to off-target viral mCherry expression.

### Chemogenetic DRN 5-HT activation during footshock-punished SA

Footshock-punished SA was evaluated in a separate group of rats from those described in the SA studies above. Prior to footshock-punished SA, rats underwent 20% EtOH SA until stabilization criteria were met. Following stabilization, rats were given 3 days of punishment testing as described previously (McElroy et al. 2023). Suppression of EtOH intake was determined by punishment resistance score, calculated as reinforcers during punishment / average reinforcers during the last 3 unpunished SA sessions (Marchant et al. 2013). A punishment resistance score of 1.0 indicates an equal number of reinforcers earned during punishment and baseline SA. Rats were pretreated with either CNO (2 mg/kg, i.p.) or vehicle 30 min prior to each punishment session.

### Data analysis

Statistical analyses were conducted using GraphPad Prism (Dotmatics, San Diego, CA). Excitability curves for DRN 5-HT neurons were analyzed through 2- or 3-way repeated measures ANOVAs followed by post hoc Sidak comparisons when appropriate. Other membrane characteristics for electrophysiology experiments in EtOH-naïve rats were analyzed by nested 1-way factorial ANOVAs with cells recorded from one rat nested within each rat, followed by post hoc Tukey’s comparisons when appropriate. Membrane characteristics in EtOH-exposed rats were analyzed by nested t-tests. IHC cell quantification was analyzed with unpaired t-tests. Behavioral data such as reinforcers earned and punishment resistance scores were analyzed by pre-planned t-tests or 1- or 2-way factorial or repeated measures ANOVAs followed by post hoc Sidak or Tukey comparisons when appropriate. Pre-planned paired t-tests were used to determine the effect of Gq DREADDs on SA compared to their responding prior to or after DREADD activation. To examine the population distribution of punishment phenotypes for rats in EtOH- and sucrose-punished SA, unbiased 2-step cluster analyses were conducted using IBM SPSS Statistics (IBM Corporation, Armonk, NY). Data were considered statistically significant at *p* < 0.05. Data are presented as mean ± SEM.

## Results

### Electrophysiological recordings in EtOH-naïve GH and SIS male and female rats

A total of 28 DRN 5-HT neurons were recorded from 19 male or female rats in adulthood following GH or SIS. Immunohistochemical verification of recorded DRN 5-HT neurons is visualized in Fig. 1b. Figure 1c-e show the effects of SIS on DRN 5-HT neuron excitability in EtOH-naïve rats via escalating current pulses (0pA to 160pA, 500ms duration) during whole-cell current-clamp recordings. Three-way RM ANOVA of AP frequency in all 4 experimental groups showed a significant effect of current (F [8,192] = 245.50, *p* < 0.0001), stress (F [1,24] = 8.56, *p* < 0.01), as well as current x stress (F [8,192] = 3.59, *p* < 0.001), sex x stress (F [1,24] = 7.23, *p* < 0.05), and current x sex x stress (F [8,192] = 3.91, *p* < 0.001) interactions (Fig. 1c). There was no significant main effect of sex nor a current x sex interaction. To further evaluate this sex x stress interaction, we ran a 2-way RM ANOVA comparing the hypoexcitable SIS females to their SIS male counterparts (Fig. 1d). Two-way RM ANOVA indicated a significant effect of current (F [8,96] = 89.53, *p* < 0.0001), sex (F [1,12] = 7.14, *p* < 0.05), and a current x sex interaction (F [8,96] = 3.08, *p* < 0.01). Post hoc Sidak test revealed that SIS females showed lower AP frequencies compared to SIS males at currents of 100, 120, and 160pA. We also ran a 2-way RM ANOVA of AP frequency between GH and SIS females (Fig. 1e). Two-way RM ANOVA revealed a significant effect of current (F [8,80] = 71.32, *p* < 0.0001), stress (F [1,10] = 11.40, *p* < 0.01) and a current x stress interaction (F [8,80] = 4.52, *p* < 0.001). Post hoc Sidak test showed that DRN 5-HT neurons in SIS females were less excitable than GH females at currents of 60-160pA.

To complement our excitability data, we evaluated certain DRN 5-HT neuron membrane characteristics in all 4 experimental groups outlined in Table 1a. Nested 1-way ANOVAs were performed with each of the cell characteristics listed in Table 1a to evaluate potential sex and/or stress effects. Our analyses revealed a main effect of group on AP threshold (F [3,15] = 3.922, *p* < 0.05) and the change in voltage from RMP to AP threshold (F [3,15] = 5.141, *p* < 0.05) but no other membrane characteristics. Post hoc Tukey’s tests show that female SIS rats have a more depolarized AP threshold than their GH female counterparts (*p* < 0.05) (Supplementary Fig. 1a) as well as a greater voltage change from RMP to AP threshold compared to SIS males and to their GH female counterparts (both *p* < 0.05) (Supplementary Fig. 1b). The changes in AP threshold are consistent with the lower excitability in SIS females compared to other groups.

**Table 1:** Membrane characteristics of DRN 5-HT neurons

	RMP (mV)	Input Resistance (MΩ)	AP Threshold (mV)	Δ AP Threshold - RMP (mV)	Latency to AP Threshold (ms)	AP Amplitude (mV)	AP Duration (ms)	AP Rise Time (ms)	AP Decay Time (ms)
a) Naïve									
Naïve GH ♀	−57.52 ± 5.41	485.07 ± 64.05	−28.13 ± 1.02	29.39 ± 5.10	172.70 ± 14.11	80.03 ± 3.80	1.77 ± 0.22	0.52 ± 0.07	1.25 ± 0.17
Naïve SIS ♀	−72.57 ± 4.21	502.15 ± 33.49	−22.20 ± 1.47 #	50.38 ± 2.97 # $	388.12 ± 59.82	79.76 ± 1.45	1.83 ± 0.06	0.50 ± 0.03	1.33 ± 0.04
Naïve GH ♂	−62.57 ± 3.40	575.13 ± 70.70	−26.10 ± 0.93	36.46 ± 3.49	285.74 ± 21.73	80.44 ± 1.93	2.05 ± 0.08	0.58 ± 0.03	1.48 ± 0.06
Naïve SIS ♂	−58.40 ± 2.74	546.59 ± 85.10	−26.96 ± 1.19	31.44 ± 3.18	317.19 ± 46.92	82.03 ± 1.29	2.41 ± 0.16	0.69 ± 0.05	1.73 ± 0.14
b) EtOH									
EtOH GH ♀	−62.67 ± 7.73	512.10 ± 98.47	−23.72 ± 1.37	38.95 ± 7.74	262.26 ± 33.24	80.11 ± 3.00	2.00 ± 0.16	0.62 ± 0.04	1.38 ± 0.12
EtOH SIS ♀	−55.72 ± 2.29	506.84 ± 77.42	−25.15 ± 1.32	30.57 ± 2.83	249.44 ± 22.59	80.15 ± 0.70	2.14 ± 0.19	0.66 ± 0.07	1.48 ± 0.14
Low EtOH Intake	−55.42 ± 4.67	591.33 ± 60.98	−23.87 ± 0.63	31.55 ± 4.80	213.67 ± 11.46	81.85 ± 1.46	1.98 ± 0.07	0.62 ± 0.03	1.37 ± 0.05
High EtOH Intake	−64.86 ± 6.77	386.68 ± 92.10	−25.28 ± 2.25	39.58 ± 7.56	319.13 ± 13.19**	77.55 ± 2.60	2.20 ± 0.29	0.68 ± 0.09	1.53 ± 0.22

Values represent mean ± standard error

# p < 0.05 vs GH females by post hoc Tukey tests

$ p < 0.05 vs SIS males by post hoc Tukey tests

** *p* < 0.01 vs low EtOH intake by Nested t-test

Total cells in EtOH-naïve group *n*=28 [GH females (6), SIS females (6), GH males (8), SIS males (8)]; Total cells in EtOH group n=10 [Stress split: GH females (5), SIS females (5); EtOH intake split: lower drinking (6), higher drinking (4)]

*RMP *resting membrane potential, *AP *action potential, *GH *group housed, *SIS *social isolation stress, *Δ* change

### Electrophysiological recordings in EtOH-exposed GH and SIS female rats

Figure 2 evaluates the effects of SIS and a history of EtOH consumption on DRN 5-HT neuron excitability, specifically in stress-vulnerable females (McElroy et al. 2023). An experimental timeline is shown in Fig. 2a. A total of 10 DRN 5-HT neurons were recorded from 8 female rats following either SIS or GH and an extensive history of EtOH consumption, including homecage drinking, SA, and punished SA (McElroy et al. 2023). The amount of EtOH consumed by this cohort was calculated for the average of the last 3 days of homecage drinking, the average of the last 3 days of SA and the average of the three punished SA sessions. The GH group consumed 5.60 ± 0.56 g/kg/24hrs in the homecage drinking model, 0.25 ± 0.07 g/kg in the SA model, and 0.27 ± 0.09 g/kg in punished SA. The SIS group consumed 4.27 ± 1.23 g/kg/24hrs in the homecage drinking model, 0.20 ± 0.05 g/kg in the SA model and 0.09 ± 0.02 g/kg in punished SA. In this small sample (*n* = 4 SIS and *n* = 4 GH), EtOH intake in homecage drinking, SA and punished SA models did not differ between treatment groups by unpaired t-test. Two-way RM ANOVA of AP frequency revealed a significant effect of current (F [8,64] = 132.70, *p* < 0.0001) but no significant effect of stress on DRN 5-HT neuron excitability (Fig. 2b). This is contrary to our findings in EtOH-naïve female rats (Fig. 1). Due to the absence of a stress effect, we collapsed across stress groups and instead divided the rats using a median-split of the average EtOH intake (g/kg body weight) during their last 3 days of unpunished EtOH SA. This analysis elicited 2 groups: higher EtOH drinking (mean: 0.32 g/kg EtOH, *n* = 4 rats) and lower EtOH drinking (mean: 0.15 g/kg EtOH, *n* = 4 rats). Two-way RM ANOVA indicated a significant effect of current (F [8,64] = 146.90, *p* < 0.0001) and EtOH intake (F [1,8] = 6.90, *p* < 0.05) as well as a current x EtOH intake interaction (F [8,64] = 2.21, *p* < 0.05) (Fig. 2c). Post hoc Sidak test showed that higher drinking females showed hypoexcitable DRN 5-HT neurons at a current of 160 pA. However, the correlation between EtOH intake and action potential frequency at a current of 160 pA did not reach statistical significance (R^2^ = 0.1768, *p* = 0.22). As we did with our EtOH-naïve electrophysiological analyses, we then evaluated DRN 5-HT neuron membrane characteristics between EtOH-exposed SIS and GH females as well as higher and lower EtOH drinking rats using nested t-tests (Table 1b). In agreement with our excitability results, we found no significant differences in membrane characteristics between GH and SIS females. However, we did find that higher EtOH-consuming females showed significantly longer latency to reach AP threshold following current stimulation (t (5) = 5.953, *p* < 0.01) (Supplementary Fig. 1b). These results, combined with EtOH-naïve data from Fig. 1, suggest that SIS and EtOH intake each diminish the excitability of DRN 5-HT neurons in females.

### Quantification of c-Fos + and Tph2 + labeled neurons within DRN subdivisions in EtOH-exposed GH and SIS female rats: impact of early life stress and punishment phenotype

An experimental timeline is shown in Fig. 2a. Figure 2e shows a 20x magnification photomicrograph of c-Fos+ (green), Tph2+ (red), and DAPI-labeled (blue) cells within the DRD, DRV, and DRVL subdivisions. Figure 2f shows a higher magnification image from the DRVL showing single-labeled Tph2 + neurons (red arrowheads), single-labeled c-Fos + neurons (green arrowheads), and Tph2+/c-Fos + double-labeled neurons (white arrowheads). Due to the functional heterogeneity of the DRN (Andrade and Haj-Dahmane 2013; McDevitt et al. 2014; Okaty et al. 2019), analyses were conducted separately for each DRN subdivision using two-tailed unpaired t-tests and compared across experimental groups (Table 2) for the effects of both stress- (Table 2a) and EtOH-punishment phenotypes (Table 2b). When comparing the total number of Tph2-positive neurons, SIS rats showed significantly lower Tph2-positive neurons than GH rats in the DRD (t (9) = 2.78, *p* < 0.05) and DRV (t (9) = 3.88, *p* < 0.01) subregions (Table 2a). Analysis of the DRVL subregion showed no significant differences between stress groups. Because brains were collected for c-Fos analysis 90 min following the final punishment session, we compared c-Fos activation patterns based on the behavioral responsivity of rats to punished SA. We conducted an unbiased 2-step cluster analysis of their punishment resistance score (see Materials and Methods) on the final day of EtOH punished SA, which revealed a bimodal distribution of punishment-resistant (mean punishment resistance score: 0.84, *n* = 7) and punishment-sensitive rats (mean punishment resistance score: 0.54, *n* = 4). There were no significant differences in our chosen neuronal endpoints between punishment phenotypes within the DRV and DRVL subregions. However, the analysis of the total number of c-Fos-positive neurons in the DRD was significantly higher in punishment-resistant rats compared to the punishment-sensitive group (t (9) = 2.48, *p* < 0.05) (Table 2b). All other DRD analyses showed no significant differences between punishment phenotypes. Our results highlight early life stress differences and DRN subdivision-specific activity differences during punished EtOH SA. We also discovered punishment phenotype differences in the DRD subdivision, alluding to a potential role of the DRD in punishment-resistant EtOH responding.

**Table 2:** Quantification of c-Fos+ and Tph2+ immunohistochemistry in EtOH-exposed female rats

	Total c-Fos + cells	Total Tph2 + cells	Total c-Fos+ & Tph2 + cells	% of c-Fos + cells that are Tph2+	% of Tph2 + cells that are c-Fos+
a) EtOH: Stress Effects					
GH Rats					
DRD	37.44 ± 7.65	110.60 ± 11.79	7.36 ± 1.55	23.08 ± 5.10	6.51 ± 0.88
DRV	45.65 ± 9.73	260.06 ± 7.01	21.07 ± 5.83	46.20 ± 5.59	8.05 ± 2.15
DRVL	40.61 ± 7.32	113.15 ± 12.71	4.03 ± 0.92	11.41 ± 2.71	3.46 ± 0.67
SIS Rats					
DRD	38.47 ± 9.69	65.13 ± 10.95*	6.50 ± 1.00	18.92 ± 3.10	11.84 ± 3.21
DRV	43.20 ± 15.46	192.40 ± 17.34**	13.93 ± 6.03	29.32 ± 8.26	7.00 ± 2.85
DRVL	30.63 ± 5.56	113.27 ± 11.02	2.83 ± 0.37	11.76 ± 3.72	2.58 ± 0.34
b) EtOH: Punishment-Resistance					
Punishment-Sensitive Rats					
DRD	22.58 ± 3.68	94.67 ± 12.49	5.50 ± 1.04	24.84 ± 3.29	6.12 ± 1.44
DRV	28.00 ± 5.97	213.25 ± 16.24	11.04 ± 5.10	33.77 ± 10.08	4.93 ± 2.09
DRVL	25.92 ± 4.37	129.58 ± 6.68	3.04 ± 0.39	13.76 ± 4.09	2.38 ± 0.36
Punishment-Resistant Rats					
DRD	46.67 ± 6.90#	87.23 ± 15.58	7.81 ± 1.26	19.10 ± 4.34	10.54 ± 2.35
DRV	53.99 ± 11.43	238.48 ± 19.01	21.70 ± 5.53	41.24 ± 6.40	9.09 ± 2.23
DRVL	41.88 ± 6.21	103.85 ± 10.97	3.74 ± 0.83	10.31 ± 2.52	3.45 ± 0.57

Values are mean ± standard error

* *p* < 0.05, ** *p* < 0.01 vs GH controls by unpaired t-tests

# *p* < 0.05 vs punishment-sensitive rats by unpaired t-test

Total rats *n*=11 [Stress split: GH rats (6), SIS rats (5); Punishment phenotype split: punishment-sensitive (4), punishment-resistant (7)]

*GH *group housed, *SIS *social isolation stress, *DRD *dorsal, dorsal raphe nucleus, *DRV *ventral, dorsal raphe nucleus, *DRVL* ventrolateral, dorsal raphe nucleus, Δ change

### Quantification of c-Fos + and Tph2 + labeled neurons within DRN subdivisions in sucrose-exposed GH and SIS female rats: impact of early life stress and punishment phenotype

Next, we conducted the same functional neuroanatomy studies in subjects exposed to a natural reward: 10% sucrose SA. Table 3 compares the effects of stress (Table 3a) and sucrose-punishment phenotypes (Table 3b) on c-Fos activation within the serotonergic DRN following punished sucrose SA through c-Fos expression. Similar to EtOH-exposed rats, sucrose-exposed rats also showed that SIS rats had a significantly lower number of Tph2-positive neurons in the DRD compared to GH rats (t (8) = 3.93, *p* < 0.01). SIS rats also had a significantly lower number of Tph2 and c-Fos colocalized neurons compared to GH controls in both the DRD (t (8) = 2.82, *p* < 0.05) and DRVL (t (8) = 2.50, *p* < 0.05) subdivisions. This stress effect also held true for the percentage of all c-Fos-positive cells that were Tph2-positive in both the DRD (t (8) = 4.86, *p* < 0.01) and the DRVL (t (8) = 3.17, *p* < 0.05). Additionally, the DRVL subregion of SIS rats also showed a significantly lower percentage of Tph2-positive cells that were c-Fos positive compared to GH rats (t (8) = 2.63, *p* < 0.05). No significant effects of stress were found in the DRV subregion (Table 3a). To assess the punishment phenotypes in sucrose rats, an unbiased 2-step cluster analysis of their punishment resistance score (see Materials and Methods) on the final day of punished sucrose SA revealed a bimodal distribution of punishment-resistant (mean punishment resistance score: 1.13, *n* = 4) and punishment-sensitive rats (mean punishment resistance score: 0.66, *n* = 6). We found no significant differences in our endpoints between punishment phenotypes (Table 3b). Although we did not see expression differences in sucrose punishment resistance phenotypes as we did with EtOH, this sucrose study further points to the DRD as an important brain region that responds to punished SA.

**Table 3:** Quantification of c-Fos+ and Tph2+ immunohistochemistry in sucrose-exposed female rats

	Total c-Fos + cells	Total Tph2 + cells	Total c-Fos+ & Tph2 + cells	% of c-Fos + cells that are Tph2+	% of Tph2 + cells that are c-Fos+
a) Sucrose: Stress Effects					
GH Rats					
DRD	69.96 ± 16.67	110.75 ± 9.11	36.04 ± 10.77	50.58 ± 5.40	40.00 ± 6.80
DRV	80.50 ± 15.00	163.29 ± 24.84	46.04 ± 12.59	54.24 ± 6.73	26.56 ± 3.94
DRVL	118.17 ± 34.41	95.00 ± 16.15	31.33 ± 10.36	25.88 ± 2.25	30.94 ± 7.31
SIS Rats					
DRD	50.18 ± 10.18	64.25 ± 7.52**	11.13 ± 1.94*	23.75 ± 2.80**	18.44 ± 3.44
DRV	74.25 ± 16.29	215.58 ± 16.20	50.54 ± 14.25	63.10 ± 6.81	21.97 ± 5.15
DRVL	51.65 ± 10.08	83.61 ± 9.08	8.76 ± 3.04*	14.88 ± 2.39*	10.69 ± 4.09*
b) Sucrose: Punishment-Resistance					
Punishment-Sensitive Rats					
DRD	47.25 ± 12.82	70.65 ± 11.21	11.35 ± 2.97	25.37 ± 5.01	17.49 ± 5.25
DRV	75.99 ± 12.24	209.54 ± 22.76	49.35 ± 19.73	60.15 ± 7.48	22.45 ± 6.80
DRVL	94.10 ± 27.87	91.92 ± 5.57	9.15 ± 2.16	16.22 ± 1.63	10.22 ± 2.57
Punishment-Resistant Rats					
DRD	65.32 ± 12.42	90.99 ± 13.40	27.58 ± 8.67	40.56 ± 6.97	27.44 ± 4.90
DRV	77.90 ± 22.83	184.75 ± 21.71	48.33 ± 10.59	59.16 ± 6.95	24.71 ± 3.94
DRVL	54.50 ± 8.20	85.67 ± 13.48	23.56 ± 8.63	21.32 ± 3.78	24.50 ± 7.21

Values are mean ± standard error

* *p* < 0.05, ** *p* < 0.01 vs GH controls by unpaired t-tests

Total rats *n*=10 [Stress split: GH rats (4), SIS rats (6); Punishment phenotype split: punishment-sensitive (4), punishment-resistant (6)]

*GH *group housed, *SIS *social isolation stress, *DRD *dorsal, dorsal raphe nucleus, *DRV *ventral, dorsal raphe nucleus, *DRVL *ventrolateral, dorsal raphe nucleus, Δ change

### Chemogenetic effects of DRN 5-HT neuron stimulation on EtOH and sucrose SA in GH female rats

Figure 3 shows the effect of chemogenetic activation of 5-HT DRN neurons on EtOH and sucrose SA. An experimental timeline is shown in Fig. 3a. Figure 3b shows the average reinforcers earned through multiple SA sessions of escalating concentrations of EtOH. One-way RM ANOVA revealed a significant effect of EtOH concentration on reinforcers earned (F [4,16] = 3.53, *p* < 0.05). Post hoc Tukey test highlighted significant differences between EtOH responding at 10% compared to both 30% and 40% EtOH concentrations. This dose-effect curve has an inverted U shape with 10–30% EtOH on the ascending limb, 30–40% at the peak, and 50% on the descending limb. Figure 3c and d show the effect of Gq-DREADD activation of 5-HT DRN neurons on self-administered EtOH concentrations on the ascending (20%) and descending (50%) limbs of the DEC. Figure 3c shows that CNO significantly reduced 20% EtOH responding acutely via pre-planned paired t-test (t (3) = 4.71, *p* < 0.05). Similarly, Fig. 3d shows that CNO significantly reduced 50% EtOH responding via pre-planned paired t-tests (t (3) = 4.24, *p* < 0.05), which then returned to baseline responding the next day (t (3) = 4.66, *p* < 0.05). To further evaluate the effect of chemogenetic activation of DRN 5-HT neurons on the consumption of a natural reward, rats underwent 20% sucrose SA. Two-tailed paired t-test of sucrose responding showed that Gq-DREADD activation significantly reduced sucrose reinforcers earned (t (3) = 23.26, *p* < 0.001) (Fig. 3e). To consider the possibility of non-specific reductions in overall responding produced by chemogenetic activation of this system, inactive lever pressing (ILP) data for the experiments represented in Fig. 3c-e was compared and found not to be impacted by chemogenetic activation. For 20% EtOH, the ILP average was: pre-CNO: 3.00 ± 0.91, CNO: 3.25 ± 1.44, post-CNO: 12.50 ± 7.22 (F (2, 6) = 1.352, *p* = 0.33). For 50% EtOH, the ILP average was: pre-CNO: 2.25 ± 0.85, CNO: 2.00 ± 0.82, post-CNO: 2.50 ± 1.55 (F (2, 6) = 0.1169, *p* = 0.89). For 20% sucrose, the ILP average was: pre-CNO: 3.50 ± 1.32, CNO: 3.25, 1.31, t (3) = 0.1093, *p* = 0.92). Together, these data indicate that chemogenetic activation of DRN 5-HT neurons reduced responding to low and high concentrations of EtOH as well as natural reward.

### Chemogenetic effects of DRN 5-HT neuron stimulation on footshock-punished EtOH SA in GH and SIS female rats

Figure 4 examines the effects of SIS and chemogenetic activation of DRN 5-HT neurons on footshock-punished SA over 3 punishment sessions. An experimental timeline is shown in Fig. 4a. Responding during punishment was assessed using punishment resistance score (see Materials and Methods). Two-way RM ANOVA of punishment resistance scores averaged over 3 days of punishment showed a significant effect of stress (F [1,14] = 9.85, *p* < 0.01) and a stress x CNO interaction (F [1,14] = 7.62, *p* < 0.05) (Fig. 4b). Post hoc Sidak test showed that SIS rats treated with CNO showed significantly more punishment-resistant responding compared to their vehicle controls as well as CNO-treated GH rats. Figure 4c evaluates punishment responding during each day of punished SA between all 4 experimental groups. As with our results in Figs. 3 and 4b, 3-way RM ANOVA revealed a significant main effect of stress (F [1,14] = 9.85, *p* < 0.01) and a stress x CNO interaction (F [1,14] = 7.62, *p* < 0.05). Multiple 2-way RM ANOVAs were performed to expand upon the significant effect of stress and the stress x CNO interaction. Without CNO treatment, GH and SIS rats show similar punishment responding over time. However, when each vehicle group is compared to their CNO stress counterparts, there is divergence in the effect of CNO on punishment. There is a main effect of CNO in both GH (F [1,5] = 15.98, *p* < 0.05) and SIS rats (F [1,9] = 6.94, *p* < 0.05). CNO resulted in a reduction in punishment responding in GH rats and an elevation of punishment responding in SIS rats. These results show stress-dependent deviations in response to chemogenetic DRN 5-HT neuron stimulation during punished SA.

## Discussion

This study takes a novel approach to determine a potential mechanism responsible for EtOH-motivated behaviors in stress-vulnerable populations through modulation of 5-HT, a sexually dimorphic neurotransmitter system involved in stress-reward interactions (Forster et al. 2018). This work expands on prior research in our laboratory that showed enhanced vulnerability of female rats to increased EtOH consumption following adolescent social isolation stress (McElroy et al. 2023). The current study used electrophysiology and functional neuroanatomy strategies to show that SIS induces persistent hypofunction of the DRN 5-HT system in females. Preliminary studies indicate that chemogenetic excitation of DRN 5-HT neurons reduced EtOH and sucrose reward value and induced stress-dependent changes in punished responding for EtOH, a model of drug consumption despite negative consequences. Collectively, these findings implicate the DRN 5-HT system in behavioral responses motivated by both rewarding and aversive stimuli.

Ex vivo electrophysiology recordings of DRN 5-HT neuron excitability revealed that SIS females showed significant deficits in DRN 5-HT neuron excitability compared to SIS males. This complements our behavioral results (McElroy et al. 2023), which showed that females were more vulnerable to SIS-induced EtOH consumption than males. Female SIS-induced hypoexcitability and subsequent elevations in EtOH consumption support prior work showing an inverse relationship between 5-HT and EtOH intake in this group (Kirby et al. 2011; Lu et al. 1994; Sari et al. 2011). In contrast, previous work in mice showed SIS-induced DRN 5-HT neuron hypoexcitability in males but hyperexcitability in females (Oliver et al. 2020; Sargin et al. 2016). In addition to species differences in these studies, electrophysiology conditions were also different, such that excitability differences in mice were elicited only following strong excitation (200–350 pA stimuli) compared to the stimulus range tested in our study (< 160 pA). In the current study, DRN 5-HT neurons from SIS females also showed more hyperpolarized RMPs, required a significantly stronger stimulus to reach AP threshold, and had a longer latency to elicit an AP following current stimulation than GH females and SIS males.

There are numerous mechanisms that may be responsible for the SIS-induced hypoexcitability of DRN 5-HT neurons we observed in our SIS females. Some of these mechanisms include enhanced inhibitory GABAergic or reduced excitatory glutamatergic tone, alterations in 5-HT_1A_ inhibitory autoreceptor sensitivity or expression, and/or changes in ionic conductance (ex: decreased I_Na_ or increased I_K_). A recent study that used a similar SIS paradigm in male mice showed SIS-induced hypoactivity of DRN 5-HT neurons but no differences in other DRN 5-HT neuron characteristics, inhibitory GABAergic afferents, or 5-HT_1A_ autoreceptor modulation between SIS and GH (Sargin et al. 2016). However, SIS mice had elevated DRN levels of the SK3 subtype of Ca^2+^-dependent K^+^ channels, blockade of which normalized the larger afterhyperpolarization potential (AHP) and excitability of 5-HT neurons of SIS mice to GH levels (Sargin et al. 2016). In addition, SIS mice in this study showed elevated anxiety- and depression-like behaviors that were also normalized by SK inhibition. Consistent with this finding, polymorphisms of the gene encoding the SK3 channel in humans have been associated with neuropsychiatric disorders characterized by emotional dysregulation, including schizophrenia, bipolar disorder, and anorexia nervosa (Chandy et al. 1998; Grube et al. 2011; Koronyo-Hamaoui et al. 2007). Though we did not evaluate the 5-HT AHP in our current study, SIS-induced upregulation of SK3 channels could be a potential mechanism to explain the less excitable 5-HT neurons in our SIS females, a hypothesis to be tested in future studies.

Next, we evaluated DRN 5-HT neuron excitability in EtOH-exposed female rats. Since the majority of SIS’s physiological effects in EtOH-naïve rats were seen in females and preclinical studies in our laboratory and others have shown the vulnerability of female rats to elevated EtOH intake (Bertholomey and Torregrossa 2019; Logrip et al. 2018; McElroy et al. 2023; Pirino et al. 2022) as well as higher sensitivity to stress-induced alcohol consumption in female clinical populations (Peltier et al. 2019; Rodriguez et al. 2020), we focused on this population for electrophysiology studies in EtOH-exposed rats. In contrast to data from naïve subjects, we did not see a persistence of DRN 5-HT neuron hypoexcitability in EtOH-exposed SIS females compared to their GH controls. In the absence of a stress effect in this cohort, we collapsed by stress conditions and performed a median-split to categorize these rats into higher or lower EtOH drinking groups based on their drinking over the last three self-administration sessions. We found that higher EtOH-consuming rats showed hypoexcitability of DRN 5-HT neurons compared to those in the lower drinking group, though the correlation between individual drinking levels and neuronal excitability did not reach statistical significance. These results partially support the inverse relationship between 5-HT neurotransmission and EtOH consumption (Burnett et al. 2012; Sachs et al. 2014). It is possible that while SIS produces DRN 5-HT hypoexcitability and initiates accelerated EtOH consumption, the impact of chronic EtOH itself may have an overall greater effect than the initial stress exposure in reducing DRN 5-HT excitability.

Acute EtOH increases extracellular 5-HT levels (Belmer et al. 2016; Kaneyuki et al. 1995; Selim and Bradberry 1996; Yan et al. 1996; Yoshimoto et al. 1992) but also reduces 5-HT neuron excitability (Maguire et al. 2014; Thielen et al. 2001), possibly via activation of presynaptic 5-HT_1A_ autoreceptors (Brodie et al. 1995). Chronic EtOH decreases extracellular 5-HT levels (Belmer et al. 2016; Kirby et al. 2011; Thielen et al. 2004; Weiss et al. 1996) but effects on 5-HT neuronal excitability are less consistent. Withdrawal from chronic EtOH has been shown to produce either hypoexcitability (Pistis et al. 1997), possibly via hypersensitivity of 5-HT_1A_ autoreceptors (Belmer et al. 2016; Scaplen and Petruccelli 2021), or hyperexcitability of DRN neurons via reduced GABAergic IPSCs and elevated glutamatergic EPSCs at different withdrawal timepoints (Lowery-Gionta et al. 2015). However, differences in the chronic regimen employed, the withdrawal timepoint chosen for recordings and lack of confirmation of the serotonergic identity of the recorded DRN neurons make these studies difficult to compare. Future studies will be needed to examine the mechanism by which moderate EtOH consumption, as modeled in our study, produces 5-HT hypoexcitability, and if this mechanism is similar or distinct from SIS-induced 5-HT hypoexcitability. Taken together, our data indicate that SIS and EtOH independently produce hypofunction of DRN 5-HT neurons. We hypothesize that SIS initiates a maladaptive positive feedback loop of 5-HT hypofunction and increased EtOH consumption which further exacerbates the neuronal phenotype, promoting continued excessive drinking.

We then evaluated DRN anatomy and activity following EtOH- and sucrose-punished SA in females. Following the final punished SA session, we employed functional neuroanatomy to capture the engagement of the serotonergic DRN system. Our results yielded stress- and subregion-specific effects on Tph2-immunoreactivity in both our EtOH-exposed and sucrose-exposed rats. Following both EtOH and sucrose exposure, SIS rats showed a reduction in Tph2 + cells in the DRD in both EtOH and sucrose groups and also in the DRV in the EtOH group compared to GH controls. Therefore, SIS-induced deficits in DRD Tph2 + cells are consistent regardless of EtOH or sucrose exposure. DRN subdivisions have distinct efferent projections. For example, the DRD projects to subcortical structures (e.g., VTA, CeA, hippocampus) (Lowry et al. 2008), whereas the DRV projects largely to cortical structures (e.g., PFC, orbitofrontal cortex) (Bang and Commons 2012). Together, immunohistochemistry and electrophysiology studies indicate that SIS in females may have reduced 5-HT synthesis and neuronal excitability, impacting a range of 5-HT targets implicated in both stress and reward responses.

To determine DRN activity during footshock-punished SA we evaluated c-Fos expression in both 5-HT and non-5-HT neurons. DRN c-Fos activation by punished EtOH SA was similar between stress groups, consistent with behavioral data showing similar punished EtOH SA in GH and SIS subjects (see vehicle-treated controls in Fig. 4b). When stress groups were pooled and instead stratified by punishment phenotype, we discovered elevated c-Fos expression in punishment-resistant rats compared to punishment-sensitive rats in the DRD, but no difference in c-Fos+/Tph2 + neurons, indicating that more non-5-HT cells are activated in punishment-resistant rats. We found no correlation between the number of shocks received during the final punishment day and c-Fos + cells in DRN subdivisions; therefore, we have reason to believe DRN neuronal activity is shock-independent. In the DRD, 75% of non-5-HT neurons contain glutamate (VGLUT3+) and project to the ventral tegmental area and substantia nigra compacta (Hioki et al. 2010). The non-5-HT c-Fos expression observed in punishment-resistant rats could be indicative of increased glutamatergic drive to reward circuits, promoting EtOH consumption despite negative consequences compared to punishment-sensitive rats. Following footshock-punished sucrose SA, SIS rats showed significantly less activation of 5-HT neurons in the DRD and DRVL compared to GH controls, though behavioral responses to punishment were similar (SIS: 0.67 ± 0.10; GH: 0.77 ± 0.26). These data may reflect SIS-induced hyporesponsiveness of the 5-HT system that may be independent of the behavioral phenotype in the sucrose punishment model.

Lastly, chemogenetic methods were employed using a within-subjects design as a preliminary evaluation of the relationship between DRN 5-HT signaling and behavior in the SA and punished SA models. We focused these studies on GH females to evaluate these behavioral responses without the added variable of ELS. Our functional neuroanatomy data and prior literature (Abumaria et al. 2007; Barton et al. 2008; Gardner et al. 2009; Lukkes et al. 2013) demonstrate that SIS induces region-specific changes in Tph2 expression and engagement in the context of EtOH consumption (Sachs et al. 2014). DRN 5-HT neurons are known to modulate the evaluation of reward value through projections to forebrain regions (e.g., medial prefrontal cortex, orbitofrontal cortex) with functions related to reinforcement learning and decision-making (Luo et al. 2015, 2016). Therefore, we first conducted a dose-effect curve for EtOH responding in order to help interpret the effects of chemogenetic activation of 5-HT neurons in this context. We found that 30–40% EtOH elicited the highest number of reinforcers in female Tph2-iCre rats on a Sprague-Dawley background. Chemogenetic activation of DRN 5-HT neurons diminished responding by approximately 60% at both 20% and 50% EtOH concentrations on the ascending and descending limbs of the dose-effect curve, respectively, consistent with a reduction in reward value. Next, we evaluated DRN 5-HT activation in the context of natural reward through 20% sucrose SA. Similarly, chemogenetic activation of DRN 5-HT neurons reduced responding for sucrose, indicating a role for the DRN 5-HT system in natural as well as drug reward.

These preliminary chemogenetic data are consistent with prior pharmacological studies in both male rats (Lu et al. 1994), primates (LeMarquand et al. 1994b) and humans (LeMarquand et al. 1994a), indicating that elevated extracellular 5-HT levels reduce EtOH intake. Other laboratories using both optogenetics and chemogenetics have reported mixed effects of DRN 5-HT stimulation on reward-related behaviors (Browne et al. 2019; Li et al. 2025; Liu et al. 2014; McDevitt et al. 2014; You et al. 2016), though some have recapitulated our finding of a reduction in reward value (McDevitt et al. 2014; You et al. 2016). It is important to note that these studies only use male rodents (Browne et al. 2019; Li et al. 2025; You et al. 2016) or fail to analyze sex as a biological variable (Liu et al. 2014; McDevitt et al. 2014). Therefore, the sex of the animals could be a potential confound contributing to the divergence in results.

The DRN 5-HT system is known to have a role in the modulation of both rewarding as well as aversive behaviors in different animal models (Cohen et al. 2015; Hayashi et al. 2015; Luo et al. 2015). Fiber photometry studies in male rats have shown that DRV projections to the orbitofrontal cortex respond to reward but not punishment, while DRD projections to the CeA respond to both reward and punishment (Ren et al. 2018). Therefore, we wanted to evaluate how chemogenetic activation of DRN 5-HT neurons would affect EtOH-punished SA, particularly in females. We have previously shown that SIS females show more punishment-resistant EtOH intake than SIS males (McElroy et al. 2023), though, in that study, we did not evaluate punishment in GH females. In the current study, vehicle-treated GH and SIS females showed similar punished EtOH SA responding. Upon chemogenetic activation of DRN 5-HT neurons, SIS rats showed increased punishment-resistant responding compared to both CNO-treated GH and to vehicle-treated SIS rats. These SIS-dependent results may reflect the hypoexcitable DRN 5-HT phenotype of SIS animals, which may make them more behaviorally responsive to exogenous chemogenetic activation.

Previous studies in our laboratory and others showed that stressors can inhibit DRN 5-HT neurons indirectly via the stress neurohormone CRF, which acts at CRF_1_-Rs on GABAergic afferents (Kirby et al. 1995, 2000, 2008; Price et al. 1998; Price and Lucki 2001). These DRN CRF_1_-Rs also mediate footshock stress-induced 22 kHz ultrasonic vocalizations (USVs): distress calls that are indicative of negative affective-like states (Li et al. 2021). Chemogenetic DRN 5-HT activation in SIS females may counteract the inhibitory effects of CRF that are induced by footshock, also reducing the negative affective response to that stressor. By reducing the “aversiveness” of the stressor, these animals may be more sensitive to the EtOH reward, promoting the punishment-resistant phenotype.

Some caveats to the chemogenetic studies include the possibility that chemogenetic activation of DRN 5-HT neurons may impact pain sensation (Berger et al. 2009; Sommer 2004) by attenuating the “painfulness” of the footshock and facilitating punishment responding. To avoid the potential confound of footshock we could use an alternative punishment model like quinine adulteration. This paradigm would allow us to investigate whether footshock-resistant rats are also quinine aversion-resistant. Because of the modest sample sizes and exclusive focus on females, our chemogenetic behavioral studies should be considered preliminary in nature and will need to be replicated with larger cohorts and in both sexes to confirm the conclusions. Additionally, this within-subjects experimental design will need to be replicated in future studies with a between-subjects design to compare CNO-treated DREADD-expressing animals both to vehicle controls and to CNO-treated non DREADD-expressing controls. Despite these limitations, we were able to see statistically significant and reversible suppression of EtOH reward value and stress-dependent changes in punished responding for EtOH as a result of chemogenetic activation of DRN 5-HT neurons.

Collectively, these data, combined with previous behavioral studies (McElroy et al. 2023), support enduring inhibitory effects of ELS on the DRN 5-HT system that are paralleled by elevated EtOH consumption, particularly in females. Additionally, our chemogenetic studies indicate a relationship between DRN 5-HT signaling and acute responses to rewards and punishers. Broadly, these data support the role of the 5-HT system in motivated behaviors across both short and long time-scales (Cohen 2015; Cohen et al. 2015).

## Supplementary information

Below is the link to the electronic supplementary material.

ESM 1 (DOCX 93.3 KB)

## Data Availability

The experimental data that support the findings of this study are available in Dryad at the following URL: 10.5061/dryad.h9w0vt4tb.

## Declarations

All procedures were conducted in accordance with the policies set forth by Temple University Institutional Animal Care and Use Committee and the National Research Council’s *Guide for the Care and Use of Laboratory Animals.*
