# Supplementary material for: Exploring the effects of adolescent social isolation stress on the serotonin system and ethanol-motivated behaviors
Source: Psychopharmacology (Berl). 2025 Feb 4;242(4):763–81. doi: 10.1007/s00213-025-06749-3 (PMC11890253; doi:10.1007/s00213-025-06749-3)
Supplement: Supplementary file 1 — (DOCX 93.3 KB) [file 213_2025_6749_MOESM1_ESM.docx]

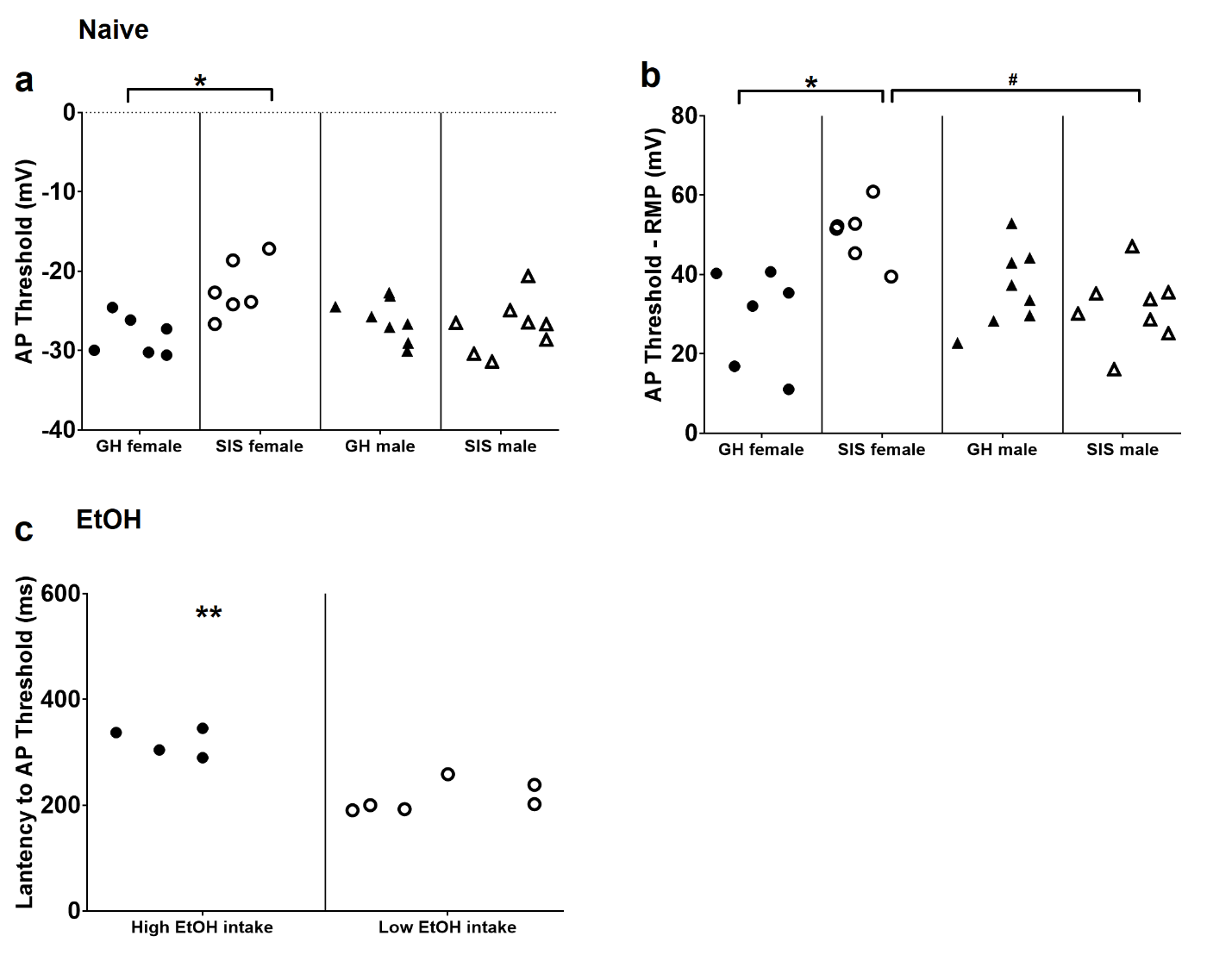


Figure 1. DRN 5-HT neuron membrane characteristics in naïve and EtOH-exposed rats. In naïve rats, female SIS rats have a) a more depolarized AP threshold than GH female counterparts (* p<0.05) as well as b) a greater voltage change from RMP to AP threshold compared to SIS males (# p<0.05) and to their GH female counterparts (* p<0.05). c) In EtOH-exposed rats, high EtOH intake females showed significantly longer latency to reach AP threshold than their low EtOH intake counterparts (** p<0.01).
